# Down-regulation of Laminin and its Correlated Significance to Interstitial Cells of Cajal in Hirschsprung's Disease

**DOI:** 10.2174/011574888X379509250805102539

**Published:** 2025-08-12

**Authors:** Yaru Mou, Dongming Wang, Jing Gao, Jian Wang, Qinghao Li

**Affiliations:** 1 Department of Postdoctoral Programme, The Affiliated Taian City Central Hospital of Qingdao University, Tai'an, China;; 2 Department of Cardiology, Shandong Provincial Hospital Affiliated to Shandong First Medical University, Jinan, Shandong, China;; 3 Department of Pediatric Surgery, Qilu Hospital, Shandong University, Jinan, China;; 4 Department of Stomatology, The Affiliated Taian City Central Hospital of Qingdao University, Tai'an, China;; 5 Department of Pediatric Surgery, The Affiliated Taian City Central Hospital of Qingdao University, Tai'an, China

**Keywords:** Laminin, interstitial cells of cajal, hirschsprung's disease, extracellular matrix, neurodevelopment, apoptosis

## Abstract

**Introduction:**

Hirschsprung’s Disease (HSCR) is characterized by aganglionosis in the distal gut, but the role of Extracellular Matrix (ECM) components in its pathogenesis remains unclear. This study investigated the relationship between laminin, a key ECM protein, and Interstitial Cells of Cajal (ICC) in HSCR.

**Methods:**

Immunofluorescence staining was used to analyze the expression and localization of laminin and ICC in paraffin-embedded colon sections from HSCR patients. Whole-mount preparations and confocal microscopy were employed to visualize the ICC network. Laminin and c-Kit expression levels were evaluated by Western blot and qPCR. Isolated ICCs were treated with laminin-targeting siRNA or exogenous laminin protein. The effects on c-Kit expression, cell viability, and apoptosis were assessed *via* Western blot, qRT-PCR, MTT assay, and TUNEL staining.

**Results:**

Laminin and ICCs were localized in the muscle layers and intermuscular regions, with laminin partially colocalizing with ICCs. In HSCR colon segments, laminin and ICC expression were significantly reduced, and ICC networks were disrupted (*p* < 0.05). Silencing laminin decreased c-Kit expression, ICC viability, and increased apoptosis, whereas exogenous laminin restored c-Kit expression, enhanced viability, and reduced apoptosis (*p* < 0.05).

**Discussion:**

Our findings suggest laminin deficiency contributes to ICC loss in HSCR, impairing intestinal motility. This aligns with prior ECM-neural crest cell studies but contrasts with reports of elevated laminin in whole-tissue analyses, possibly due to regional or temporal differences. Limitations include reliance on rodent ICC models.

**Conclusion:**

Laminin supports ICC viability and prevents apoptosis. Reduced laminin expression in HSCR contributes to the loss of ICC, disrupting pacemaker activity and impairing colonic motility.

## 1 INTRODUCTION

Hirschsprung's Disease (HSCR) is a congenital disorder characterized by the absence of ganglion cells in the distal gut. It has an incidence of approximately 1 in 5,000 live births and manifests clinically as delayed meconium passage and distal intestinal obstruction in neonates, as well as chronic constipation in older children [1]. Despite numerous studies and various surgical modifications, such as the Transanal Endorectal Pull-Through (TEPT) procedure, since its initial description by Harald Hirschsprung in 1886 [2, 3], the exact pathogenesis of HSCR remains poorly understood.

Several studies have highlighted the crucial role of the intestinal microenvironment in the pathogenesis of HSCR, demonstrating that interactions with the Extracellular Matrix (ECM) are essential for the migration of Enteric Neural Crest-Derived Cells (ENCDCs) [4]. Additionally, a reduction in Interstitial Cells of Cajal (ICC) has been observed in the pathological intestinal segments of HSCR patients, with ICC being closely associated with enteric nerves. This reduction in ICC may contribute to the intestinal dysfunction observed in HSCR [5]. However, the underlying mechanisms responsible for the decrease in ICC remain unclear, and no studies have yet explored the interaction between intestinal microenvironmental factors and ICC.

In this study, we aimed to investigate the correlation between laminin expression and the variation in ICC in HSCR. We hypothesized that the ECM microenvironment may influence the pacemaker cells of the distal colon in HSCR, thereby contributing to the pathophysiology of the disease.

## 2 MATERIALS AND METHODS

### 2.1 Patients and Sample Preparation

Between April 2018 and June 2020, 28 children (12 boys and 16 girls) diagnosed with HSCR at the Department of Pediatric Surgery, Qilu Hospital, Shandong University, were enrolled in this study. The age range of these patients was 4 to 16.4 months (median age: 10.7 months). All diagnoses were pathologically confirmed based on the absence of ganglion cells in the distal colon. For each patient, distal aganglionic, proximal ganglionic, and transitional colon segments were obtained from surgically resected specimens. Mucosa and submucosa were carefully removed under a dissecting microscope, and each segment was divided for subsequent Western blot, qPCR, and histological or immunofluorescent analyses.

All procedures involving human participants and animals were reviewed and approved by the Ethics Committee of Qilu Hospital, Shandong University (Approval No. KYLL-2017(KS)-012). Informed consent was obtained from the legal guardians of all minor participants during preoperative consultations. Human tissue samples used in this study were derived from surgically excised specimens, and their collection did not pose any additional harm to the participants. All animal experiments were conducted in strict accordance with the institutional guidelines for animal care and use, as approved by the same ethics committee.

### 2.2 Western Blot Analysis

Total protein was extracted from each colon sample using the Minute™ Total Protein Extraction Kit (Invent, USA), and protein concentrations were determined by the BCA method. Protein samples (30 μg) were loaded onto a 7.5% SDS-PAGE gel and electroblotted onto PVDF membranes. After blocking with 5% BSA for 1 hour, the membranes were incubated overnight at 4°C with rabbit anti-human laminin antibody (1:1000, Abcam, USA), mouse anti-human c-kit antibody (1:1000, Abcam, USA), and mouse anti-human β-actin antibody (1:1000, CST, USA). Following washing with 0.1M TBST, the membranes were incubated with HRP-conjugated secondary antibodies (1:5000, CST, USA) for 1 hour at room temperature. After further washing, protein signals were detected using the ECL system, and the gray values of laminin, c-kit, and β-actin were quantified. Each experiment was repeated at least three times.

### 2.3 Quantitative PCR Assay

Total RNA was extracted from the specimens using the MiniBEST Universal RNA Extraction Kit (TaKaRa, Japan). RNA concentration was assessed, and cDNA was synthesized from 1 μg of RNA using the PrimeScript™ RT Reagent Kit (TaKaRa, Japan). Quantitative PCR reactions were performed using the UltraSYBR Mixture (Cwbio, China) on a Roche LightCycler 480 system. Primer sequences are listed in Table **1**. Ct values were determined, and relative gene expression was calculated using the 2−ΔΔCt method.

### 2.4 Immunofluorescence Staining

Colon samples were fixed for 6 hours and embedded in paraffin. Sections (3 μm) were prepared, de-waxed in xylene, and rehydrated through graded alcohols. Antigen retrieval was performed by microwaving the slides in 0.01 M citrate buffer. After rinsing with 0.1 M PBS, the sections were blocked with goat serum for 30 minutes at 37°C and then incubated overnight at 4°C with rabbit anti-human laminin antibody (1:100, Abcam, USA) and mouse anti-human c-kit antibody (1:100, Abcam, USA). The sections were then incubated with goat anti-mouse Alexa 488 and goat anti-rabbit Alexa 594 antibodies (1:200, Abcam, USA) at room temperature in the dark for 2 hours. After washing with PBS, sections were mounted with DAPI-containing hard-set mounting medium, and fluorescence images were captured using DAPI, FITC, and Texas Red filter sets. For whole-mount visualization, fixed colon samples were blocked with blocking buffer (5% goat serum, 0.1% Triton X-100 in PBS) for 2 hours at 37°C, and then incubated overnight at 4°C with mouse anti-human c-kit antibody (1:100, Abcam, USA) in blocking buffer. The samples were then incubated with goat anti-mouse Alexa 488 (1:200) at room temperature for 2 hours, rinsed with PBS, and mounted for confocal microscopy imaging. Whole-mount confocal imaging was performed using a Zeiss LSM710 laser scanning confocal microscope. Z-stack images were acquired at 1-µm intervals and processed using ZEN 3.1 software (Carl Zeiss).

### 2.5 Isolation and Culture of ICC

Five timed-pregnant Sprague-Dawley (SD) rats (gestational day 18, female, weight not limited) were used for embryonic tissue collection. A total of 16 rats were used in this study. All procedures were approved by the Ethics Committee of Qilu Hospital, Shandong University (KYLL-2017(KS)-012). The rats were anesthetized using pentobarbital, and embryos were harvested under sterile conditions. Embryonic colons were excised, luminal contents flushed with Krebs–Ringer solution, and mucosal layers were carefully removed under a dissecting microscope.

Muscle strips were incubated in calcium-free Hanks' solution for 30 minutes, then digested using an enzyme solution containing 2 mg/mL collagenase (Sigma, USA), 2 mg/mL bovine serum albumin (Gibco, USA), 2% antibiotics (Sigma, USA), and 0.27 mg/mL ATP (Sigma, USA). The cell suspension was filtered through a #200-mesh sieve and incubated with a FITC-labeled c-Kit antibody (1:50 dilution, Santa Cruz, USA) for 30 minutes at 4°C. After labeling, cells were incubated with anti-FITC microbeads (Miltenyi, 130-048-701) at a 1:10 bead-to-cell ratio. Magnetic cell separation was performed using LS columns (Miltenyi) under a 0.5 T magnetic field. Flow-through cells were discarded, and c-Kit-positive cells were eluted after column removal. Purified cells were cultured in DMEM/F12 medium (Gibco, USA) supplemented with 10% fetal bovine serum (Gibco, USA), 2% B27 supplement (Gibco, USA), 5 ng/mL stem cell factor (R&D Systems, USA), and 2% antibiotics (Sigma, USA). Cultures were maintained at 37°C in a humidified atmosphere with 5% CO_2_.

### 2.6 Identification and Interventions

ICC was identified immunologically using mouse anti-rat c-kit antibody (1:100, Abcam, USA) and rabbit anti-rat α-smooth muscle actin antibody (1:100, Abcam, USA). Small interfering RNA (siRNA) transfection (GenePharma, China) was performed using Lipofectamine™ 2000 Transfection Reagent (Invitrogen, USA) to silence laminin expression. The siRNA sequences are provided in Table **2**. Recombinant rat laminin protein (Gibco, USA) was used for exogenous intervention at concentrations of 10 μg/mL (group LN1) and 20 μg/mL (group LN2). After treatment, laminin and c-kit expression were assessed *via* Western blot and quantitative PCR. The primary antibodies used were rabbit anti-rat laminin antibody (1:800, Abcam, USA), mouse anti-rat c-kit antibody (1:600, Abcam, USA), and mouse anti-rat β-actin antibody (1:1000, CST, USA).

### 2.7 MTT Assay

Cells were seeded in 96-well plates at a density of 5,000 cells per well and treated with siRNA or exogenous laminin. Cell viability was measured at 6, 12, 24, 36, and 48 hours post-seeding using the MTT Cell Proliferation/Cytotoxicity Assay Kit (Beyotime, China). MTT (5 mg/mL) was added to each well and incubated for 4 hours at 37°C with 5% CO_2_. After incubation, the medium was removed, and the formazan crystals were dissolved in 150 μL DMSO. Absorbance at 490 nm was measured to assess cell viability. Each test was repeated at least three times.

### 2.8 TUNEL Assay

Cells were seeded in 24-well plates and treated with siRNA and exogenous laminin (20 μg/mL) with or without TNF-α (30 ng/mL, R&D, USA). Apoptosis was assessed using the TUNEL assay (In-Situ Cell Death Detection Kit, Roche, Mannheim, Germany) according to the manufacturer's instructions. After 48 hours of treatment, the cells were fixed with 4% paraformaldehyde for 30 minutes, permeabilized with 0.5% Triton X-100, and incubated with TUNEL reaction solution for 1 hour in the dark. After staining with DAPI and adding an anti-fluorescence quencher, apoptosis was quantified under a fluorescence microscope with excitation wavelengths of 570–620 nm.

### 2.9 Statistical Analysis

Data were analyzed using GraphPad Prism^®^ 7.0 software and presented as mean ± SD. One-way ANOVA was used for multiple comparisons, with p-values less than 0.05 considered statistically significant.

## 3 RESULTS

### 3.1 Expression of Laminin and ICC in the Colon of HSCR Patients

**Fig. (1):**
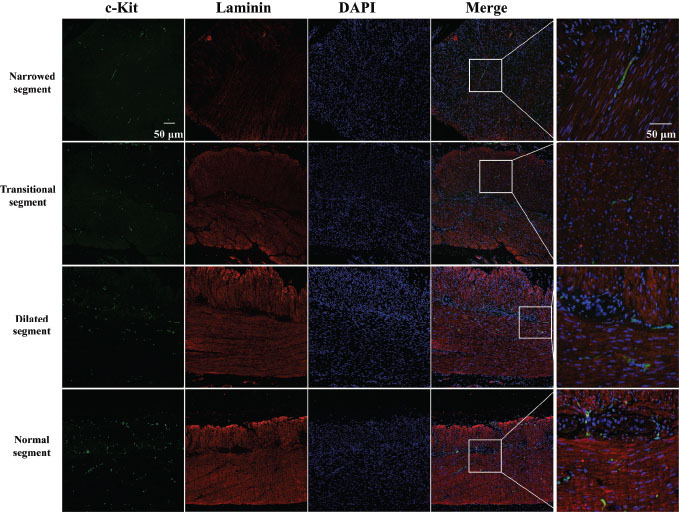
Immunofluorescence staining of Laminin and ICC in segments of colon with HSCR. c-Kit-positive ICC cells showed green fluorescence, and laminin showed red fluorescence. They were observed in circular muscle layers, longitudinal muscle layers, and between them, with decreased expression in the narrowed segment and partly overlapping.

**Fig. (2):**
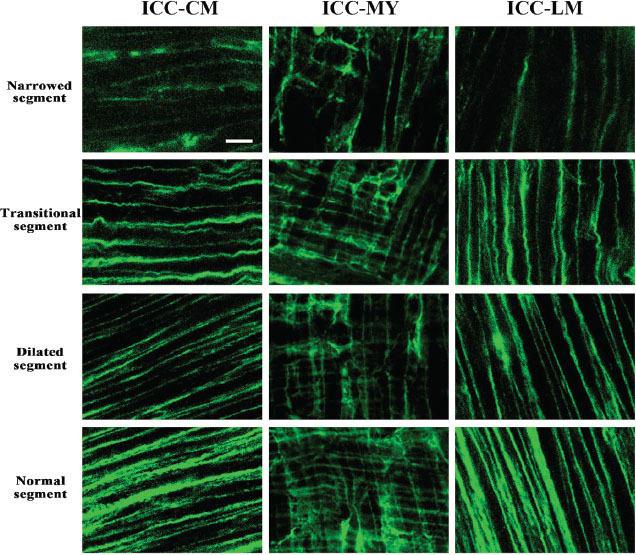
Disruption of ICC networks in HSCR-affected colon segments visualized by whole-mount immunofluorescence staining. Confocal microscopy images of ICC networks labeled with c-Kit (green) in the circular muscle layer (ICC-CM), longitudinal muscle layer (ICC-LM), and myenteric layer (ICC-MY). Compared to non-affected regions, the narrowed aganglionic segments exhibited fragmented ICC networks and diminished spatial continuity. Scale bars: 50 μm.

**Fig. (3):**
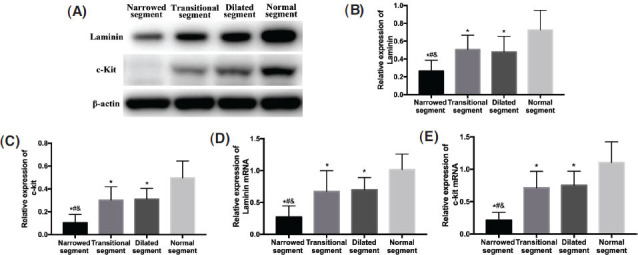
The expressions of Laminin and c-Kit in segments of the colon with HSCR. Representative blots (**A**), comparisons of relative gray values (**B**, **C**) in Western-blot analysis, and Relative expressions in Quantitative PCR assay (**D**, **E**) showed that the expressions of Laminin and c-Kit decreased markedly in the narrowed segment (*p* < 0.05). (* *p* < 0.05 *vs.* normal segment, # *p* < 0.05 *vs* transitional segment, and *p* < 0.05 *vs.* dilated segment).

Immunofluorescence staining was used to investigate the histological distribution of Laminin and Interstitial Cells of Cajal (ICC) in colon segments affected by Hirschsprung's Disease (HSCR). ICC, identified by c-Kit antibody labeling, were observed in the Circular Muscle layer (ICC-CM), Longitudinal Muscle layer (ICC-LM), and Myenteric layers (ICC-MY). The abundance of ICC was significantly reduced in the narrowed segments of the colon from HSCR patients (Fig. **1**). Whole-mount immunofluorescence staining revealed that ICC networks were markedly disrupted in the narrowed segments of the colon compared to other segments (Fig. **2**). Laminin expression was detected in the basement membrane of the colon and similarly decreased in the narrowed regions, corresponding with the reduced ICC abundance (Fig. **1**). Both Western blot analysis and quantitative PCR (qPCR) confirmed the downregulation of Laminin and c-Kit expression at both protein and mRNA levels (*p* < 0.05, Fig. **3**). Co-localization of ICC and Laminin in the colon segments revealed partial overlap, suggesting that Laminin is expressed in the ICC basement membrane and may interact with ICC (Fig. **1**).

### 3.2 Correlation Between Downregulated Laminin Expression and ICC Dysfunction

**Fig. (4):**
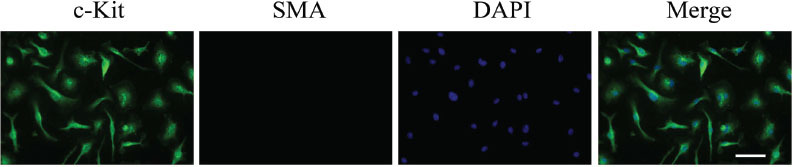
Immunofluorescence validation of purified ICC after selective culture. The ICCs were isolated from rat colon *via* enzymatic dissociation and enriched using magnetic-activated cell sorting with c-Kit-specific microbeads. Purified cells were immunostained for c-Kit (green, ICC marker) and α-Smooth Muscle Actin (SMA, red, smooth muscle cell marker). Nuclei were counterstained with DAPI (blue). The absence of the SMA signal confirms the exclusion of smooth muscle cells, demonstrating > 95% purity of the cultured ICC population. Scale bar: 50 μm.

**Fig. (5):**
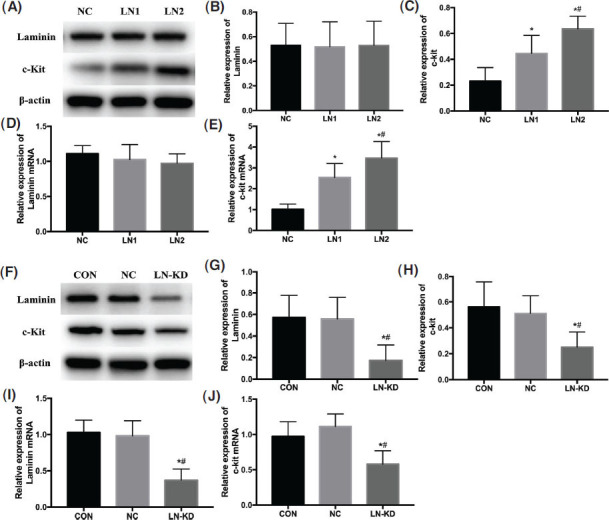
The expression of c-Kit after interventions. Western blot analysis and Quantitative PCR assay were used to detect the changes in laminin and c-Kit after interventions. It was found that exogenous Laminin had no significant effect on the expression level of endogenous Laminin in ICC, but it could up-regulate the expression of c-Kit (**A**-**E**) in a dose-dependent manner. The increase in the LN2 group (20 ug/ml) was more significant than that of the LN1 group (10 ug/ml) (*p < 0.05 *vs* NC group, #*p* < 0.05 *vs* LN1 group). After silencing Laminin expression by siRNA transfection, the expression of c-Kit decreased with the decrease of Laminin expression level in ICC cells (**F**-**J**) (*p < 0.05 *vs.* CON group, #*p* < 0.05 *vs.* NC group). LN-KD: laminin knockdown; NC: negative control; CON: control; LN1: 10 μg/ml Laminin; LN2: 20 μg/ml Laminin.

The ICC was isolated from the colon of 18-day pregnant SD rats through enzymatic dissociation and enriched using magnetic-activated cell sorting. Immunofluorescence staining confirmed that the isolated cells expressed c-Kit and lacked α-Smooth Muscle Actin (SMA) (Fig. **4**). Initially, recombinant rat Laminin was added to the culture medium at doses of 10 µg/ml (group LN1) and 20 µg/ml (group LN2), and the expression of c-Kit was measured. Exogenous Laminin did not alter endogenous Laminin levels but significantly upregulated c-Kit expression in a dose-dependent manner (*p* < 0.05; Figs. **5A**-**C**), with mRNA expression showing a consistent trend (Figs. **5D**, **E**). Conversely, silencing Laminin expression using siRNA led to a significant decrease in c-Kit expression (*p* < 0.05; Figs. **5F**-**H**), with corresponding mRNA levels also showing a similar downward trend (Figs. **5I**, **J**).

**Fig. (6):**
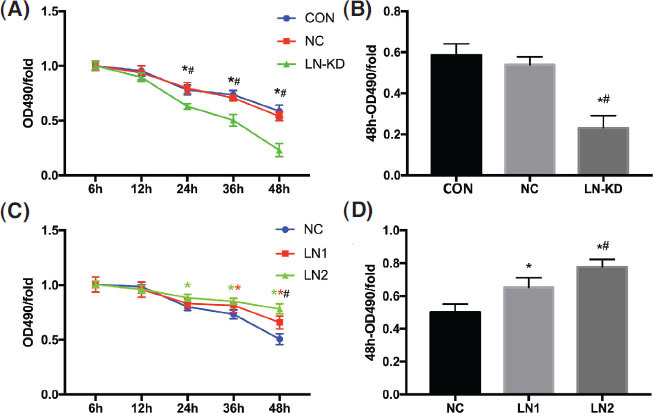
The effects of Laminin on the survivals of ICC in the MTT assay. After siRNA transfection silenced the Laminin expression of ICC cells, ICC in the LN-KD group showed a more significant decrease in cell activity starting from 24h after the intervention (**A**, **B**). (*p < 0.05 *vs* CON group, #*p* < 0.05 *vs* NC group). Exogenous rat laminin could inhibit the decline of ICC cell activity, and showed a dose dependence (**C**, **D**). The LN2 (20 ug/ml) group showed a statistical difference from 24 hours after intervention. The LN1 (10 ug/ml) group showed a statistical difference at 36 hours after intervention. At 48 hours after intervention, the LN2 group had a more obvious inhibition effect on the decrease of ICC cell activity than the LN1 group (*p < 0.05 *vs.* CON group, #*p* < 0.05 *vs.* NC group). LN-KD: laminin knockdown; NC: negative control; CON: control; LN1: 10 μg/ml Laminin; LN2: 20 μg/ml Laminin.

**Fig. (7):**
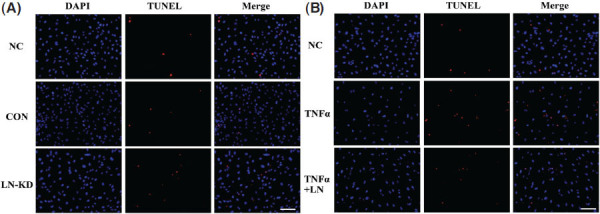
The effects of Laminin on the apoptosis of ICC in the TUNEL assay. TUNEL staining showed that the apoptosis of the LN-KD group was increased after siRNA silencing of laminin expression in ICC (**A**). Since ICC had less apoptosis under normal conditions, TNF-α was used to induce ICC cell apoptosis. The apoptosis of ICC was increased after TNF-α induction, and the increase in apoptosis of ICC was inhibited when exogenous laminin (20 µg/ml) and TNF-α were added into the medium at the same time (**B**). LN-KD: laminin knockdown; NC: negative control; CON: control. Scale bar: 50 μm.

To assess the impact of Laminin on ICC survival, we performed MTT assays. Knockdown of Laminin *via* siRNA resulted in a significant reduction in ICC viability (*p* < 0.05, Figs. **6A**, **B**), while exogenous Laminin restored ICC survival in a dose-dependent manner (*p* < 0.05, Figs. **6C**, **D**). TUNEL assays revealed increased spontaneous apoptosis in ICC following Laminin knockdown (Fig. **7A**). Additionally, TNF-α-induced apoptosis of ICC was significantly inhibited when Laminin was added to the medium, suggesting that Laminin protects ICC from apoptosis under inflammatory conditions (Fig. **7B**).

## 4 DISCUSSION

A major hypothesis explaining the pathogenesis of HSCR suggests that abnormal expression or distribution of Extracellular Matrix (ECM) components, such as Laminin, may influence the microenvironment surrounding Enteric Neural Crest-Derived Cells (ENCDCs), potentially affecting their colonization and resulting in innervation deficiencies in HSCR. Laminins are critical ECM components that promote neurite outgrowth and neuronal survival, and have been implicated in the pathogenesis of HSCR [6-8]. As key regulators of gastrointestinal motility, ICCs act as pacemaker cells, particularly in the ICC-MY, and integrate excitatory and inhibitory signals, thus orchestrating peristaltic movements in the gut [9-11]. Previous studies have also shown a reduction or disruption of ICC networks in HSCR [11-17]. Although Laminin and ICC are both associated with HSCR, the direct relationship between them remains unclear and warrants further investigation. This study aims to address this gap by exploring the role of Laminin in ICC function, which may help explain the reduced ICC presence in HSCR-affected segments.

In this study, we observed that both Laminin and ICC were significantly reduced in the narrowed segments of the colon in HSCR patients. While our cohort included patients ranging from neonates to older children (10.7 ± 5.7 months), no significant age-dependent differences in laminin or ICC expression were observed. However, future studies with larger age-stratified cohorts are needed to confirm this finding. Our findings suggest that the decrease in ICC may be linked to reduced Laminin expression. The knockdown of Laminin expression in ICC led to decreased survival and increased apoptosis, while exogenous Laminin promoted ICC survival and inhibited apoptosis, even under inflammatory conditions induced by TNF-α. These results indicate that Laminin is essential for maintaining ICC survival in HSCR. Additionally, c-Kit, a key biomarker of ICC, was found to be upregulated by exogenous Laminin and downregulated by Laminin knockdown, further supporting the role of Laminin in regulating ICC function and suggesting that Laminin may be a key player in maintaining normal intestinal motility through its effects on ICC.

Interestingly, our findings contrast with those of some previous studies, which reported increased Laminin expression in the narrowed segments of HSCR [18]. Several factors could explain this discrepancy. First, the timing of Laminin expression may vary, as studies in a mouse model of HSCR have shown that Laminin expression fluctuates over time, with differences observed at various embryonic stages [19]. Second, differences in specimen treatment could account for the observed variations in Laminin expression. Previous studies analyzed Laminin expression in whole colon tissue, while our study focused specifically on the seromuscular layer, where ICCs are predominantly located. Additionally, distinct Laminin subtypes may be involved in different stages of HSCR, which could explain regional differences in expression patterns. Lastly, genetic variability between regions may contribute to these observed differences, as shown by regional differences in the prevalence of specific genetic mutations, such as the NRG1 gene associated with HSCR in Asia [20]. Laminin likely regulates c-Kit expression *via* integrin-mediated signaling. Mechanistically, Laminin is likely involved in the regulation of c-Kit expression through integrin-mediated signaling. Previous studies have shown that Laminin–integrin interactions activate focal adhesion kinase and downstream PI3K/Akt pathways, promoting cell survival and inhibiting apoptosis [21]. In addition, Laminin’s role in ECM mechanotransduction may facilitate the stabilization and clustering of c-Kit receptors, as demonstrated in ICC models [22]. Further investigation into these signaling mechanisms is warranted.

In conclusion, our study highlights the role of the intestinal microenvironment, particularly Laminin, in regulating ICC function and suggests that altered Laminin expression may contribute to the reduction of ICC in HSCR. This novel insight into the interplay between ECM components and ICC in HSCR opens new avenues for future research. Although laminin was the primary focus of the present study, future studies should compare its role with other ECM components, such as collagen IV and fibronectin, which also interact with ICC. Despite these advances, further studies are needed to elucidate the underlying mechanisms and provide a more comprehensive understanding of how the intestinal microenvironment influences the development and progression of HSCR. Future investigations will explore the interaction between intestinal microenvironmental factors and ICC, aiming to identify potential therapeutic targets for improving ICC function and restoring intestinal motility in HSCR patients. While the findings highlight reduced laminin in HSCR-affected colon segments, its utility as a non-invasive biomarker (*e.g*., in serum or stool) remains speculative. Future studies could explore laminin fragments (*e.g*., LN-332) in biofluids to assess their correlation with disease severity or postoperative motility outcomes. Lastly, our use of rat-derived ICCs reflects both the practical and ethical constraints associated with obtaining and culturing human ICCs. To bridge this translational gap, future efforts should focus on establishing biobanks of HSCR specimens and optimizing protocols for human ICC isolation and culture through collaborative research initiatives.

## CONCLUSION

This study demonstrates that Laminin and ICC are significantly reduced in the narrowed segments of the colon in HSCR, leading to disrupted networks and decreased c-Kit expression. Laminin supports ICC survival and inhibits apoptosis, suggesting its reduction may contribute to ICC loss and impaired intestinal motility in HSCR. These findings highlight the critical role of the intestinal microenvironment in HSCR pathogenesis.

## STUDY LIMITATIONS

This study has several limitations. First, the cohort size was relatively small, and although patients of different ages were included, age-stratified analyses with larger cohorts are needed to validate our findings. Second, discrepancies between our results and previous reports may partly reflect methodological differences, such as the specific tissue layers analyzed and the potential involvement of distinct Laminin subtypes. Third, functional experiments were conducted using rat-derived ICC rather than human ICC, due to ethical and technical constraints, which may limit the direct translational relevance of the findings. Finally, while we focused primarily on Laminin, other extracellular matrix components such as collagen IV and fibronectin may also play important roles in HSCR and warrant further investigation.

## Figures and Tables

**Table 1:** Detailed information of primers.

**Primer**	**Species**	**F Sequences (5’-3’)**	**R Sequences (5’-3’)**
Laminin	Human	GGCTTCCGTTGTCAGCAATC	TCAAGTTTCTCAGCGTTGGC
c-kit	Human	CGTTCTGCTCCTACTGCTTCG	CCCACGCGGACTATTAAGTCT
β-actin	Human	AGCGAGCATCCCCCAAAGTT	GGGCACGAAGGCTCATCATT
Laminin	Rat	GGCAGATGTATGTAGCAAGA	TAGGTCTCGTTCTCAGCAT
c-kit	Rat	AAGGAGGCTATTGAAGGTAG	AGACATTGGTGGCAACTTA
β-actin	Rat	CCACCATGTACCCAGGCATT	ACGCAGCTCAGTAACAGTCC

**Table 2:** Detailed information of siRNA sequences.

**-**	**Species**	**F Sequences (5’-3’)**	**R Sequences (5’-3’)**
Laminin	Rat	GCUGCAAACCAGGAUUCUATT	UAGAAUCCUGGUUUGCAGCTT
Negative control	Rat	UUCUCCGAACGUGUCACGUTT	ACGUGACACGUUCGGAGAATT

## Data Availability

The data and supportive information are available within the article.

## LIST OF ABBREVIATIONS

ECM: Extracellular Matrix
ENCDCs: Enteric Neural Crest-Derived Cells
HSCR: Hirschsprung’s Disease
ICC: Interstitial Cells of Cajal
SD: Sprague-Dawley
SMA: α-Smooth Muscle Actin
